# Identification of QTNs Controlling Seed Protein Content in Soybean Using Multi-Locus Genome-Wide Association Studies

**DOI:** 10.3389/fpls.2018.01690

**Published:** 2018-11-21

**Authors:** Kaixin Zhang, Shulin Liu, Wenbin Li, Shiping Liu, Xiyu Li, Yanlong Fang, Jun Zhang, Yue Wang, Shichao Xu, Jianan Zhang, Jie Song, Zhongying Qi, Xiaocui Tian, Zhixi Tian, Wen-Xia Li, Hailong Ning

**Affiliations:** ^1^Key Laboratory of Soybean Biology in the Chinese Ministry of Education, Northeast Agricultural University, Harbin, China; ^2^Northeastern Key Laboratory of Soybean Biology and Breeding/Genetics in the Chinese Ministry of Agriculture, Northeast Agricultural University, Harbin, China; ^3^State Key Laboratory of Plant Cell and Chromosome Engineering, Institute of Genetics and Developmental Biology, Chinese Academy of Sciences, Beijing, China; ^4^College of Agronomy, Jilin Agricultural University, Changchun, China

**Keywords:** protein content, soybean, multi-locus GWAS, QTNs, four-way recombinant inbred lines

## Abstract

Protein content (PC), an important trait in soybean (*Glycine max*) breeding, is controlled by multiple genes with relatively small effects. To identify the quantitative trait nucleotides (QTNs) controlling PC, we conducted a multi-locus genome-wide association study (GWAS) for PC in 144 four-way recombinant inbred lines (FW-RILs). All the FW-RILs were phenotyped for PC in 20 environments, including four locations over 4 years with different experimental treatments. Meanwhile, all the FW-RILs were genotyped using SoySNP660k BeadChip, producing genotype data for 109,676 non-redundant single-nucleotide polymorphisms. A total of 129 significant QTNs were identified by five multi-locus GWAS methods. Based on the 22 common QTNs detected by multiple GWAS methods or in multiple environments, pathway analysis identified 8 potential candidate genes that are likely to be involved in protein synthesis and metabolism in soybean seeds. Using superior allele information for 22 common QTNs in 22 elite and 7 inferior lines, we found higher superior allele percentages in the elite lines and lower percentages in the inferior lines. These findings will contribute to the discovery of the polygenic networks controlling PC in soybean, increase our understanding of the genetic foundation and regulation of PC, and be useful for molecular breeding of high-protein soybean varieties.

## Introduction

Soybean [*Glycine max* (L.) Merr.] is a globally important high-protein crop, with protein accounting for about 40% of the seed’s dry weight. Soybean is one of humans’ main sources of dietary protein; therefore, breeding high-protein varieties of soybean is an ongoing, important objective of plant breeders. The efficiency of plant breeding has been greatly accelerated by the emergence of molecular markers and molecular technology, such as random amplified polymorphic DNA, restriction fragment length polymorphism, amplified fragment length polymorphism, simple sequence repeats, specific-locus amplified fragment, and single-nucleotide polymorphism (SNP). For genome-wide association studies (GWAS) in soybean, the acquisition of a large number of molecular markers is extremely important (Song et al., 2013), and SNPs are well suited for such analyses because of their high densities throughout the genome (Gaur et al., 2012).

Breeders and molecular geneticists have routinely used populations derived from biparental crosses for development of new varieties and mapping quantitative trait loci (QTLs) for traits of interest. However, the richness of allelic and phenotypic variation in biparental inter-mated populations is somewhat limited. To overcome this limitation, animal breeders have developed the multi-parental inter-mated population design, for example by using a population descended from eight mouse strains (Yalcin et al., 2005). Recently, multi-parent advanced generation inter-cross (MAGIC) lines have also been developed in plants, including wheat (*Triticum aestivum*; Huang et al., 2012; Mackay et al., 2014), maize (*Zea mays*; Dell’Acqua et al., 2015), *Arabidopsis thaliana* (Kover et al., 2009), barley (*Hordeum vulgare*; Sannemann et al., 2015), tomato (*Solanum lycopersicum*; Pascual et al., 2015), and rice (*Oryza sativa*; Bandillo et al., 2013; Meng et al., 2016a,b). MAGIC populations have more diverse alleles than bi-parental populations, which increases genetic variation. However, obtaining a MAGIC population is labor intensive because of the repeated crossing and requires large population sizes to include recombinants for all the desirable traits. An intermediate design, the four-way cross population, is easier to obtain while providing some of the same benefits as an eight-way cross population.

In recent years, GWAS with high-density SNPs have emerged as very powerful tools for dissecting the genetic basis of complex traits. This approach has been applied to MAGIC populations of many crop plants (Bandillo et al., 2013; Pascual et al., 2015; Meng et al., 2016a,b) but not, as of yet, to soybean. As in soybean, either biparental populations or natural populations have been used in all previous QTL mapping studies (Li et al., 2018; Wang et al., 2018). This previous research indicates that PC in soybean is a typical quantitative trait controlled by multiple genes with relatively small genetic effects, whose identification will require a more efficient method to detected QTLs. Multi-locus GWAS is a suitable method for identifying significant QTNs, especially for relatively small effects; it also has a low false positive rate, and has been used in many studies (Liu et al., 2016; Wang et al., 2016; Tamba et al., 2017; Zhang Y. et al., 2017; Wen et al., 2018).

In this study, we used 144 recombinant inbred lines (FW-RILs) from a four-way cross, which were genotyped by SNPs and phenotyped seed protein content (PC) in different environments. We then combined these data to identify significant QTNs for PC in soybean using multi-locus GWAS methods. The objective was to find common QTNs that were identified by multiple methods or in multiple environments and then deduce potential candidate genes and identify elite lines in the FW-RIL population, as a means to accelerate molecular breeding to increase PC in soybean.

## Materials and Methods

### Plant Materials

Four soybean varieties, Kenfeng14 (PC 41.08%), Kenfeng15 (PC 41.42%), Kenfeng19 (PC 43.06%), and Heinong48 (PC 43.55%), were used to construct a four-way recombinant inbred line (FW-RIL) population. Among these, Kenfeng14, Kenfeng15, and Kenfeng19 were bred by the Heilongjiang Academy of Agricultural Reclamation and derived from the crosses Suinong 10 × Changnong 5, Suinong 14 × Kenjiao 9307, and Hefeng 25 × (Kenfeng 4 × Gong 8861-0), respectively; and Heinong48 was bred by Heilongjiang Academy of Agricultural Science and derived from the cross Ha 90-6719 × Sui 90-5888.

First, two single crosses, Kengfeng14 × Kenfeng15 and Kenfeng19 × Heinong48, were carried out in Harbin (45.75°N, 126.63°E), Heilongjiang Province, China, and the F_1_ seeds were harvested in 2008. Second, a cross was conducted between two sets of single-cross F_1_ seeds, and F_1_ seeds of the resulting four-way cross (Kengfeng14 × Kenfeng15) × (Kenfeng19 × Heinong48) were harvested in 2009. Third, the four-way cross F_1_ seeds were self-crossed for six generations continuously by alternate sowing in Yacheng (17.5°N, 109.00°E), Hainan Province, China, in the winter and in Harbin in the summer from 2010 to 2014, using the single-seed descent method to select single seeds from individual plants in each generation. Finally, a total of 144 FW-RILs were obtained for this study.

### Field Experiment and Phenotype Data Collection

The four parental lines and 144 FW-RILs were planted in 20 environments with different locations, years, seedling densities, fertilizers, and sowing dates. The detailed planting schedule is summarized in Supplementary Table S1. All plant materials in each environment were grown in three-row 5 m × 0.7 m plots in a completely randomized block design with three replications. The experimental plots were managed identically to local soybean crops. Ten plants from the middle of the plots for each line (four parents and 144 FW-RILs) were harvested and the seeds threshed separately for each of the 20 environments. The total PC of seeds (dry seeds, with water content of about 10%) was determined in three random samples from mixed seed of each line by the near-infrared analyzer (Infratec 1241, Foss, Denmark) at the Key Laboratory of Soybean Biology of the Chinese Education Ministry at the Northeast Agricultural University in China. The calibration regression technique was Partial Least Square (PLS), which involved combining spectral data with laboratory data (Kjeldahl method) to calculate seed PC, described by the percentage of seed weight. The phenotypic values given for each parental and FW-RIL used in this study were all the mean values of three repetitions.

### Statistical Analyses of Phenotypic Data

Mean, standard deviation, minimum, maximum, range, skewness, kurtosis, and coefficient of variation (CV) for FW-RILs in each environment were calculated. A correlation analysis between each pair of environments was performed. Analysis of variance (ANOVA) for single environment and jointly multiple environments was conducted with “varietal effect model of genotype” and “environment + genotype + environment × genotype interaction,” respectively, and the broad-sense heritability (*h*^2^) was estimated by the following equation:

$$h^{2}=\sigma_{G}^{2}/(\sigma_{G}^{2}+\sigma_{\mathrm{GE}}^{2}/e+\sigma^{2}/\mathrm{er})$$

where $\sigma_{G}^{2}$ is the genotypic variance, $\sigma_{GE}^{2}$ is the variance due to the genotype × environment interaction, σ^2^ is the error variance, *e* is the number of environments, and *r* is the number of replications within an environment. The statistical analysis was implemented by SAS 9.2 (SAS Institute, Cary, NC, United States).

### Genotyping

Juvenile leaves from parents and FW-RIL plants were collected, frozen in liquid nitrogen, and immediately ground into powder. Total genomic DNA was extracted using the CTAB method (Doyle et al., 1990) and eluted in 50 μl deionized water. The DNA concentration was determined using a UV752N spectrophotometer (Shanghai Jingke Science Instrument Co. Ltd.) and was diluted to 100 ng ± 1 ng in deionized water. SNP genotyping was performed at Beijing Boao Biotechnology Co. Ltd, using the SoySNP660K BeadChip. A total of 109,676 SNPs across 20 chromosomes remained after quality filtering; the SNP markers identified were filtered for minor allele frequency (MAF > 0.05), and the maximum missing sites per SNP was < 10% (Belamkar et al., 2016). Heterozygous loci were then marked as missing to obtain better estimates of marker effects, and the SNP markers were re-filtered using the same filtering criteria and used for the next analysis of population structure, kinship, and GWAS.

### Analysis of Population Structure and Linkage Disequilibrium

The analysis of population structure was performed with the software STRUCTURE 2.3.4 (Pritchard et al., 2000). For each run, the number of burn-in iterations was 10,000, followed by 2000 Markov chain Monte Carlo (MCMC) replications after burn-in. The admixture and allele frequencies correlated models were considered in the analysis. Ten impended iterations were used in the STRUCTURE analysis. The hypothetical number of subpopulations (K) ranged from 1 to 10. The best K was identified according to Evanno et al. (2005) using STRUCTURE HARVESTER (Earl and Vonholdt, 2012).

TASSEL 5.0 was utilized to analyze linkage disequilibrium (LD) (Bradbury et al., 2007) by analyzing *r*^2^ values of all pairs of SNPs located within 10 Mb physical distance, the LD decay trend was found following regression of the negative natural logarithm, and the physical distance of LD decay was estimated as the position where *r^2^* dropped to half of its maximum value.

### Genome-Wide Association Studies

The software mrMLM.GUI (version 3.0) was used to perform the GWAS. Five multi-locus GWAS methods within mrMLM.GUI were used to identify significant QTNs, including mrMLM (Wang et al., 2016), FASTmrMLM (Tamba et al., 2017), FASTmrEMMA (Wen et al., 2018), pLARmEB (Zhang J. et al., 2017), and ISIS EM-BLASSO (Tamba et al., 2017). The critical *P*-value parameters for these methods at the first stage were set to 0.01 except for FASTmrEMMA, where the critical *P*-value was set to 0.005, and the critical LOD score was set to 3 for significant QTN at the last stage. All these five methods involved the population structure and kinship matrices in this study, and the kinship matrix was calculated with the software mrMLM.GUI 3.0.

### Superior Allele Analysis

We considered the QTNs we detected from multiple environments or by multiple methods as common QTNs. Based on the effect values of each common QTN and the genotype for code 1, we could determine the superior alleles of each QTN. If the effect value of the QTN is positive, the genotype for code 1 is the superior allele; if the effect value is negative, another genotype is the superior allele. For each QTN, the superior allele percentage in the 144 FW-RILs was equal to the number of lines containing the superior allele divided by the total number of lines. For each line, the proportion of superior alleles in these QTNs was calculated as the number of superior alleles divided by the total number of QTNs. A heat map visualizing the percentage of superior alleles was obtained in the R (heatmap package) program (Mellbye and Schuster, 2014).

### Identification of Potential Candidate Genes

The search for potential candidate genes based on the common QTNs detected by multiple methods or in multiple environments was performed using four steps. First, the intervals that include each common QTN were selected on the Phytozome website^1^. These intervals were determined by the rate of LD decay. Second, genes highly expressed in the form process of seed protein through the Bio-Analytic Resource for Plant Biology (BAR) website^2^ were identified. Third, based on the experimental data in the Plant Expression Database (PLEXdb) website^3^, the differentially expressed genes among high and low protein lines were identified from the above high expression genes. Finally, all the differentially expressed genes were put together for analysis of pathways on the Kyoto Encyclopedia of Genes and Genomes (KEGG) website^4^, and potential candidate genes were identified by the result of pathway analysis.

## Results

### Protein Content Phenotype

**FIGURE 1:**
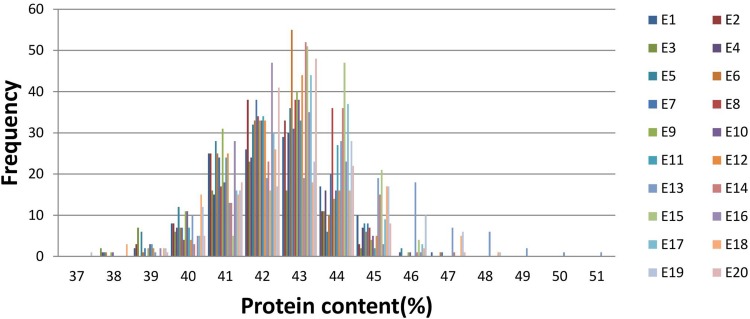
Frequency distribution of seed protein content under 20 environments.

We measured the PC phenotypes of the parents and the 144 FW-RILs in 20 environments, which are presented in Supplementary Table S2 and Supplementary Figure S1. Graphing the average value for PC of each line in 20 environments revealed that the 144 lines show extensive variation in PC (Supplementary Figure S1). Examination of the values (Supplementary Table S2) showed that the parental lines Kenfeng14 and Kenfeng15 had lower PC than Kenfeng19 and Heinong48 in all 20 environments. And the “Range” (Range = PC_Max_ – PC_Min_) of the four parents in 20 environments was from 1.70 to 4.04%; the “Range” of FW-RILs was 5.20–11.56%, representing a large difference in PC, especially in FW-RILs. Kurtosis and skewness (absolute value) were less than 1, indicating the continuous normal distribution of the PC values (Supplementary Table S2 and Figure 1). We found a high coefficient of variation in PC across all the environments. The ANOVA results of parents and FW-RILs both indicated that extremely significant variation exists in genotype, environment, and genotype-by-environment (Tables 1, 2). The mean square values for the genotype-by-environment interaction were all less than the mean square values of genotype, and the estimated broad-based heritability was high, being 85.46%. The correlation coefficients between each pair of environments were almost all positive, and many were significant or extremely significant (Supplementary Table S3), indicating high consistency across various environments.

**Table 1:** Joint ANOVA of PC of parent lines in multiple environments.

Source	DF	SS	MS	*F*	Pr > F
Replication	2	5.55	2.77	6.59	0.0018
Environment	19	155.86	8.20	19.49	<0.0001
Genotype	3	271.22	90.41	214.80	<0.0001
Genotype ^∗^ Environment	57	50.02	0.877	2.08	0.0002
Error	158	66.50	0.42		

**Table 2:** Joint ANOVA of PC of FW-RILs in multiple environments and heritability.

Source	DF	SS	MS	*F*	Pr > F	Variance component
Replication	2	1,304.62	652.31	11,639.60	<0.0001	0.25
Environment	19	1,893.98	99.681	1,778.72	<0.0001	0.23
Genotype	143	4,565.61	31.93	569.70	<0.0001	0.50
Genotype ^∗^ Environment	2,436	12,408.82	5.09	90.89	<0.0001	1.68
Error	5,196	291.19	0.06			0.06
*h*^2^						0.85

### Population Structure and LD

**FIGURE 2:**
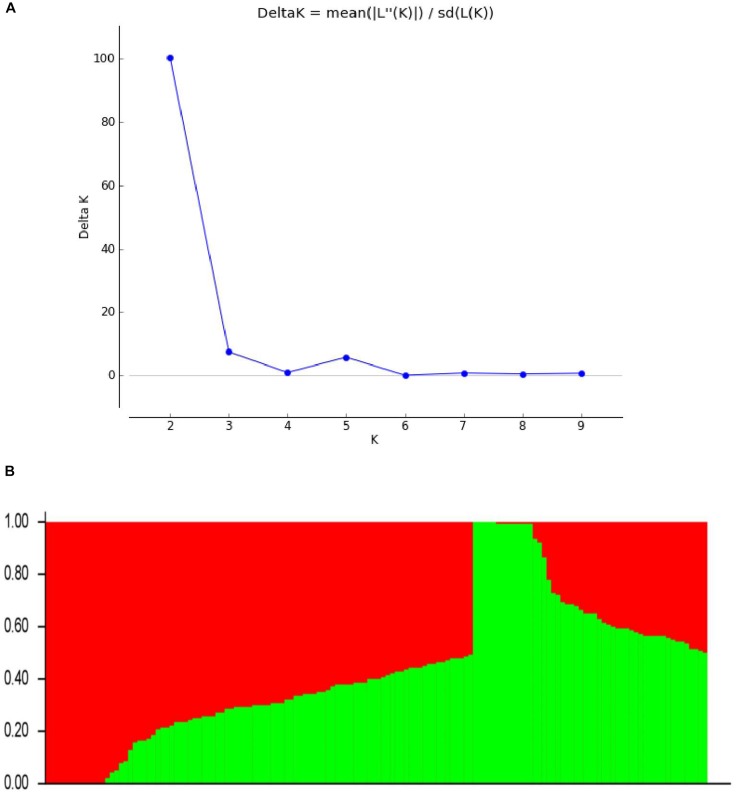
Population structure based on 5375 SNPs distributed across 20 chromosomes. **(A)** Plot of ΔK calculated for *K* = 1–10. **(B)** Population structure (*K* = 2); the areas of the two colors (green and red) illustrate the proportion of each subgroup.

To define the subpopulations within the panel of 144 lines, as described by Pritchard et al. (2009), we selected 5375 of the 109,676 SNPs that had better polymorphisms and were randomly distributed across the 20 soybean chromosomes. Delta *K* (ΔK) was calculated using STRUCTURE 2.3.4 (Figure 2A; *K* = 1–10), revealing the presence of two subpopulations (selected *K* = 2) based on ΔK values (Figure 2B). These two subgroups contained 53 (36.81%) and 91 (63.19%) lines.

We analyzed the *r^2^* values of all pairs of SNPs located within 10 Mb of each other and determined the LD decay trend based on regression to the negative natural logarithm. As shown in Supplementary Figure S2, *r^2^* decreased gradually with increased distance, and the LD decay distance was estimated at 1.2 Mb, where *r^2^* dropped to half of its maximum value (0.45). Because the population used in this study is derived from parents, the speed of LD decay is slower and the LD decay distance is much longer than that of a natural population.

### QTNs Detected by Multi-Locus GWAS Methods

**FIGURE 3:**
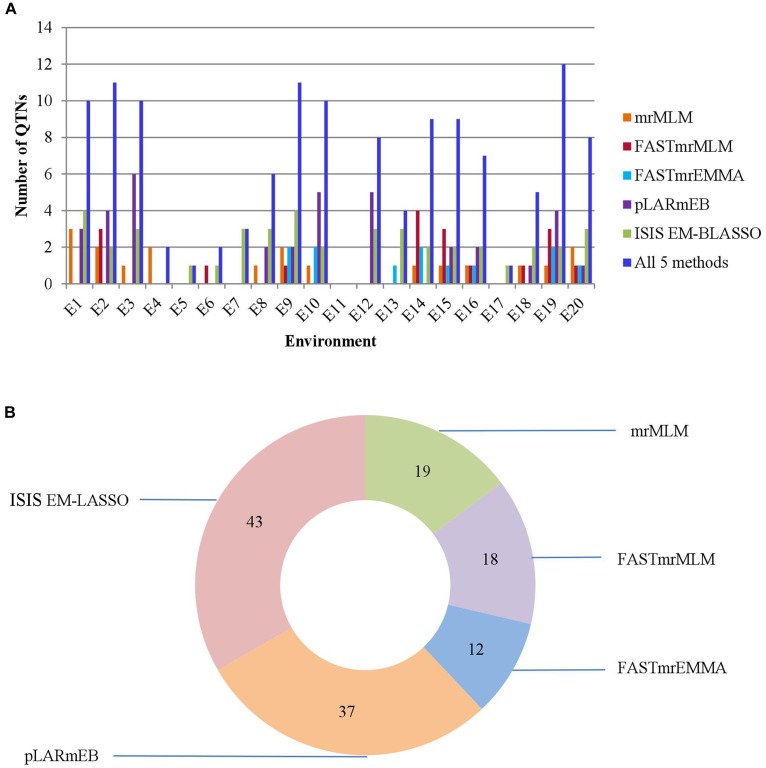
**(A)** The total numbers of significant QTNs detected in 20 environments across 5 methods. **(B)** The total numbers of significant QTNs detected using each of 5 multi-locus GWAS methods in 20 environments.

We identified 19, 18, 12, 37, and 43 significant QTNs for PC using mrMLM, FASTmrMLM, FASTmrEMMA, pLARmEB, and ISIS EM-BLASSO, respectively, and 10, 11, 10, 2, 1, 2, 3, 6, 11, 10, 0, 8, 4, 9, 9, 7, 1, 5, 12, and 8 significant QTNs, respectively, in the 20 environments (Figure 3 and Supplementary Table S4). No significant QTN was detected in the eleventh environment (E11).

We further checked the common QTNs across multiple environments. As discussed above, only one such common QTN was identified in three environments (Table 3). The single QTN (AX-157298785) was located on chromosome 18, with the LOD values ranging from 4.02 to 5.33 (Table 3). The proportion of phenotypic variance explained (PVE) by the QTN ranged from 8.40 to 11.02%, and the QTN direction of effect (positive or negative) was consistent across different environments and different methods (Table 3).

**Table 3:** Stable expressed QTNs identified in multiple environments and by multiple methods.

Method^a^	Env	Marker	Chr	Marker position	QTN effect	LOD score	*r*^2^(%)^b^
1,1	E14,E20,	AX-157298785	18	6,620,851	−0.39, −0.40,	4.21, 4.02,	10.78, 11.02,
2,3	E14,E14,				−0.29, −0.67,	4.32, 4.43,	5.86, 7.78,
5,5	E14,E16				−0.34, −0.39	4.56, 5.33	8.40, 10.48

*^a^mrMLM, FASTmrMLM, FASTmrEMMA, pLARmEB, and ISIS EM-BLASSO were indicated by 1 to 5, respectively. ^b^r^2^ (%), proportion of total phenotypic variation explained by each QTN.*

**FIGURE 4:**
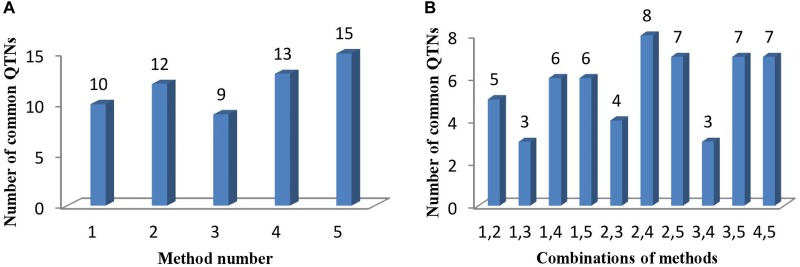
**(A)** The number of common QTNs detected by different methods and **(B)** different combinations of methods. Method numbers correspond to (1) mrMLM, (2) FASTmrMLM, (3) FASTmrEMMA, (4) pLARmEB, and (5) ISIS EM-BLASSO.

Comparing the results across the different approaches, we found that 22 common QTNs (including AX-157298785) were identified simultaneously by at least two approaches (Table 4); these were located on chromosomes 1, 2, 3, 4, 6, 7, 9, 10, 12, 14, 16, and 18. Their LOD values ranged from 3.06 to 6.90, the proportion of PVE by each QTN ranged from 3.84 to 19.21%, and the direction of effect (positive or negative) of each QTN was also consistent across the different methods (Table 4). Of the 22 common QTNs, 12, 5, and 5 were identified simultaneously by 2, 3, and 4 approaches, respectively. Among the five methods, ISIS EM-BLASSO detected the highest number of common QTNs (Figure 4A), and among the combinations of two methods, FASTmrMLM combined with pLARmEB detected the highest number of common QTNs (Figure 4B).

**Table 4:** Common QTNs for seed protein content in soybean across different multi-methods.

Method^a^	Marker	Chr	Position (bp)	QTN effect	LOD score	*r^2^* (%)^b^
2,3	AX-157088197	1	2,142,538	0.24,0.51	3.06,3.15	5.41,5.50
2,5	AX-157514742	1	5,244,469	0.41,0.44	4.76,4.76	8.58,9.99
2,4	AX-157393800	1	36,630,129	0.53,0.61	4.17,5.49	6.39,8.57
2,3,4	AX-157197609	2	19,981,350	−0.34,−0.65,−0.38	3.38,3.36,3.81	8.06,6.14,9.58
3,5	**AX-157074676**	**2**	**43,036,996**	**−0.64,−0.30**	**3.65,3.10**	**5.59,4.83**
2,4	AX-157487767	3	28,963,194	0.45,0.47	4.72,3.90	4.74,5.30
2,3,4,5	**AX-157594705**	**4**	**47,793,555**	**−0.35,−0.65,−0.42,−0.35**	**3.94,3.24,5.26,4.40**	**6.97,5.61,9.85,6.96**
1,2,4,5	**AX-157397239**	**4**	**47,801,472**	**−0.32,−0.27,−0.23,−0.25**	**3.13,3.07,3.43,3.73**	**9.42,6.54,4.75,5.88**
1,2,4,5	**AX-157489326**	**6**	**48,361,864**	**0.43,0.34,0.42,0.31**	**3.78,4.26,6.53,3.37**	**11.81,7.50,11.14,6.19**
1,4	AX-157083233	6	49,396,770	−0.78,−0.51	3.20,3.65	19.21,6.67
1,3,4,5	**AX-157462104**	**7**	**20,724,011**	**0.82,1.35,0.65,0.50**	**4.64,4.97,5.90,3.21**	**15.18,9.83,9.58,5.78**
1,2,5	**AX-157506141**	**9**	**34,120,396**	**−0.56,−0.44,−0.41**	**4.02,4.03,3.63**	**12.36,7.81,6.79**
3,5	**AX-157570733**	**10**	**43,785,659**	**0.75,0.34**	**5.09,3.88**	**9.27,7.63**
4,5	**AX-157566978**	**12**	**1,258,280**	**−0.24,−0.36**	**3.65,4.52**	**3.84,8.50**
3,5	**AX-157069070**	**12**	**10,655,900**	**0.83,0.42**	**4.24,5.50**	**8.30,8.67**
4,5	**AX-157357710**	**12**	**11,111,913**	**−0.61,−0.62**	**6.90,5.32**	**9.83,12.74**
2,4,5	**AX-157217990**	**14**	**7,160,557**	**−0.23,−0.24,−0.23**	**3.42,3.22,3.63**	**4.79,5.45,4.92**
1,4	AX-157512649	16	24,057,874	0.31,0.24	3.16,3.44	7.00,4.09
1,5	**AX-157168337**	**16**	**28,693,806**	**0.64,0.37**	**4.66,3.94**	**14.20,5.23**
1,3,5	AX-157333937	18	2,064,407	0.56,0.96,0.48	3.56,4.33,4.23	11.90,8.54,8.58
1,2,4	AX-157443296	18	6,597,875	−0.49,−0.36,−0.38	3.83,4.26,5.12	15.96,8.71,9.88
1,2,3,5	AX-157298785	18	6,620,851	−0.39(−0.40),−0.29, −0.67,−0.34 (−0.39)	4.21 (4.02),4.32, 4.43,4.56,(5.33)	10.78(11.02),5.86, 7.78,8.40 (10.48)

*^a^mrMLM, FASTmrMLM, FASTmrEMMA, pLARmEB, and ISIS EM-BLASSO were indicated by 1–5, respectively. ^b^r^2^ (%), proportion of total phenotypic variation explained by each QTN. Bold text indicates the QTNs appeared to be near QTLs associated with protein content in soybean that had been mapped in earlier studies.*

We found only one stable QTN that was identified not only in multiple environments but also by multiple methods (Table 3): AX-157298785, located on chromosome 18, which was detected by mrMLM, FASTmrMLM, FASTmrEMMA, and ISIS EM-BLASSO in environments E14, E16, and E20, with LOD values ranging from 4.02 to 5.33 and PVE values ranging from 5.86 to 11.02% (Table 3).

### Distribution of Superior Alleles in the FW-RILs

**FIGURE 5:**
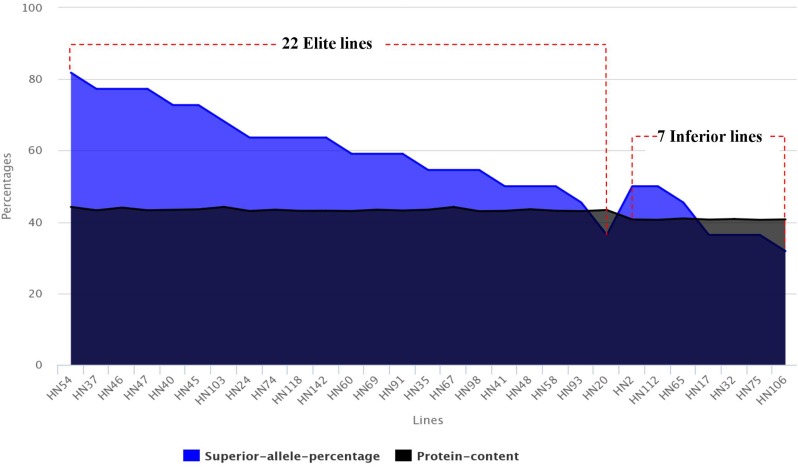
Distribution of superior allele percentages and the PC in the 29 high- and low-PC lines.

Based on the PC averages in 20 environments for each FW-RIL, we found that 22 lines had higher phenotypic values (43.07–44.21%) and 7 lines had lower phenotypic values (40.60–40.98%) (Table 5). For each of the 22 elite lines, the percentages of superior alleles (PSA) across 22 common QTNs ranged from 36 to 82% (Table 5), 91% (20 of the 22 lines) showed PSAs of ≥50%, and only 9% showed PSAs of <50%. For each of the 7 lines with lower phenotypic values, the PSAs ranged from 32 to 50% (Table 5), only 2 lines (28%) had PSAs of ≥50%, and the remaining 5 (71%) had PSAs of <50%. Thus, the elite lines with higher PC have more superior alleles than the lines with lower PC (Figure 5).

**Table 5:** Phenotypic averages of seed protein content and proportion of superior alleles in 29 lines across 22 common QTNs.

Line	PC (%)	PSA (%)	Line	PC (%)	PSA (%)
HN54	44.21	82	HN67	44.19	55
HN37	43.26	77	HN98	43.01	55
HN46	44.01	77	HN41	43.10	50
HN47	43.27	77	HN48	43.55	50
HN40	43.40	73	HN58	43.13	50
HN45	43.54	73	HN93	43.03	45
HN103	44.20	68	HN20	43.34	36
HN24	43.07	64	**HN2**	**40.69**	**50**
HN74	43.42	64	**HN112**	**40.60**	**50**
HN118	43.11	64	**HN65**	**40.98**	**45**
HN142	43.14	64	**HN17**	**40.66**	**36**
HN60	43.06	59	**HN32**	**40.81**	**36**
HN69	43.43	59	**HN75**	**40.60**	**36**
HN91	43.20	59	**HN106**	**40.71**	**32**
HN35	43.43	55			

*Bold font indicates the 7 lines with lower protein contents, and non-bold font indicates the 22 lines with higher protein contents.*

**FIGURE 6:**
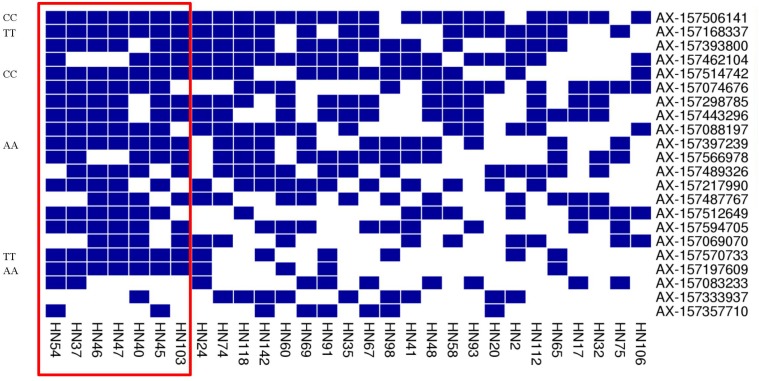
Heat map of the superior allele distribution for the 22 common QTNs in the 29 high- and low-PC lines. Blue and white colors represent superior and inferior alleles, respectively.

Based on the superior allele information for the 22 common QTNs in 29 lines, the PSAs for each QTN ranged from 36 to 95% in the 22 elite lines, with 16 QTNs showing ≥ 50% superior alleles while the remaining 6 QTNs showed < 50%. The range of PSAs for each QTN was 0–71% in the 7 lines with lower phenotypic values; 8 of the QTNs had PSAs ≥ 50% and the remaining 14 QTNs had PSAs < 50% (Table 6 and Figure 6). The number of QTNs with ≥50% superior alleles was higher in the 22 elite lines than in the 7 inferior lines. Based on these results, we can easily find elite lines by identifying superior alleles for application in breeding higher PC soybean.

**Table 6:** Superior alleles and their proportions of 22 common QTNs in 22 elite and 7 inferior lines.

QTN	Superior allele	PSA (%)^a^	PSA (%)^b^	QTN	Superior allele	PSA (%)^a^	PSA (%)^b^
AX-157506141	CC	95	71	AX-157074676	GG	64	71
AX-157514742	CC	82	29	AX-157489326	TT	59	57
AX-157168337	TT	77	57	AX-157594705	GG	59	29
AX-157393800	AA	77	43	AX-157487767	AA	50	57
AX-157462104	GG	77	43	AX-157570733	TT	50	29
AX-157298785	GG	73	43	AX-157512649	GG	45	71
AX-157397239	AA	73	29	AX-157197609	AA	45	14
AX-157443296	CC	68	57	AX-157069070	TT	41	57
AX-157088197	GG	68	43	AX-157333937	CC	41	14
AX-157566978	AA	68	43	AX-157083233	TT	36	29
AX-157217990	TT	68	14	AX-157357710	AA	36	0

*^a^Indicates the percentage of superior allele of each common QTN in 22 elite lines. ^b^Indicates the percentage of superior allele of each common QTN in 7 inferior lines.*

In addition, we found some common superior alleles in multiple elite lines: for example, the seven lines HN54, HN37, HN46, HN47, HN40, HN45, and HN103 all contained the superior alleles AX-157506141, AX-157168337, AX-157514742, AX-157397239, AX-157570733, and AX-15719760 (Figure 6), and the superior allele AX-157506141 occurred in 21 of the 22 elite lines. We suspect that common superior alleles may have a particularly strong influence on PC. In further research, we hope to make use of this information to breed better soybean using marker-assisted selection.

### Potential Candidate Genes Determined Based on Common QTNs

We used the LD decay distance to select potential candidate genes within a specific distance of each common QTN. Because of the nature of the population (i.e., derived from parents), the LD decay distance is large, so we determined the range of potential candidate genes according to the position of the fastest decay rate. In Supplementary Figure S2B, we can see that LD decays fastest before 200 kb, and then tends to flatten, so we searched for potential candidate genes in the interval of 100 kb on either side of each QTN. Following the four steps described in the Materials and Methods, a total of 288 genes were found in these intervals and 96 genes were expressed highly in seed at the form process of seed protein. Among the 96 genes, 34 genes were differentially expressed among high and low protein lines, and these 34 genes were used to do pathway analysis.

**FIGURE 7:**
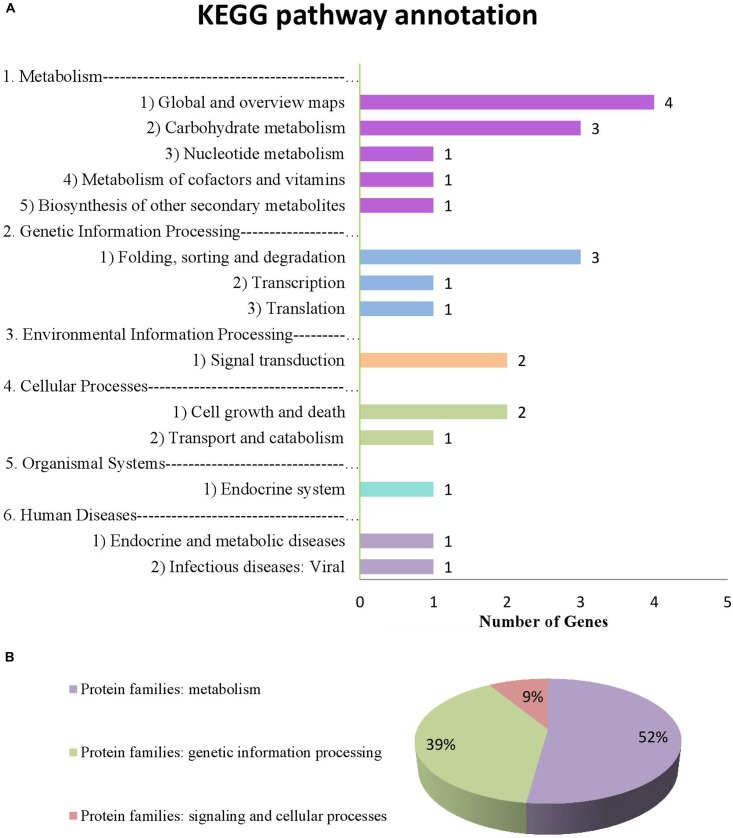
Information on pathways and orthologous protein families of 17 genes. **(A)** shows the information on pathway. **(B)** shows the information on orthologous protein families.

From the annotation data, we found that 17 of 34 genes (51.4% of the genes we submitted) were previously annotated in 14 pathways and 3 protein families in the KEGG database (Supplementary Tables S5, S6 and Figure 7). Of these, 8 were considered potential candidate genes based on the information of their annotation and functions in metabolic pathways (Table 7 bold text).

**Table 7:** Details of 17 genes annotated in the KEGG database.

QTN name	Gene name^a^	Chromosome	Position	KO number	Annotation
AX-157088197	Glyma.01G020900	chr01	2104937..2109784	K00083	E1.1.1.195; cinnamyl-alcohol dehydrogenase [EC:1.1.1.195]
AX-157514742	Glyma.01G046000	chr01	5317206..5321170	K21734	SLD; sphingolipid 8-(E/Z)-desaturase [EC:1.14.19.29]
AX-157487767	**Glyma.03G100800**	**chr03**	**28980378..28988564**	**K00948**	**PRPS; ribose-phosphate pyrophosphokinase [EC:2.7.6.1]**
AX-157397239	Glyma.04G206300	chr04	47880082..47890343	K21594	GUF1; translation factor GUF1, mitochondrial [EC:3.6.5.-]
AX-157570733	Glyma.10G207100	chr10	43860713..43863694	K01373	CTSF; cathepsin F [EC:3.4.22.41]
AX-157570733	**Glyma.10G207300**	**chr10**	**43879369..43883196**	**K16298**	**SCPL-IV; serine carboxypeptidase-like clade IV [EC:3.4.16.-]**
AX-157566978	Glyma.12G017200	chr12	1212436..1217081	K17790	TIM22; mitochondrial import inner membrane translocase subunit TIM22
AX-157566978	Glyma.12G018000	chr12	1252709..1256330	K04683	TFDP1; transcription factor Dp-1
AX-157566978	Glyma.12G018800	chr12	1321415..1326705	K1542	PPP4R2; serine/threonine-protein phosphatase 4 regulatory subunit 2
AX-157566978	**Glyma.12G019300**	**chr12**	**1354109..1356766**	**K11599**	**POMP; proteasome maturation protein**
AX-157357710	**Glyma.12G112900**	**chr12**	**11064160..11065437**	**K00793**	**ribE; riboflavin synthase [EC:2.5.1.9]**
AX-157357710	Glyma.12G113400	chr12	11135703..11140450	K19355	MAN; mannan endo-1,4-beta-mannosidase [EC:3.2.1.78]
AX-157217990	**Glyma.14G081600**	**chr14**	**7064342..7068643**	**K03030**	**PSMD14; 26S proteasome regulatory subunit N11**
AX-157333937	Glyma.18G027100	chr18	2033839..2038697	K05857	PLCD; phosphatidylinositol phospholipase C, delta [EC:3.1.4.11]
AX-157333937	**Glyma.18G028600**	**chr18**	**2154790..2160008**	**K00685**	**ATE1; arginyl-tRNA—protein transferase [EC:2.3.2.8]**
AX-157443296	**Glyma.18G071100**	**chr18**	**6667467..6671661**	**K12275**	**SEC62; translocation protein SEC62**
AX-157443296	**Glyma.18G071300**	**chr18**	**6687583..6692093**	**K12880**	**THOC3; THO complex subunit 3**

*Bold font indicates the genes which correlate with the protein anabolism in soybean according to our deduction. ^a^Indicates the gene which correlates with the QTN (before the gene in the same row).*

## Discussion

In this study, we employed multi-locus GWAS with an FW-RIL population of the MAGIC population type to identify QTNs related to PC of soybean. Twenty-two common QTNs were detected by multiple methods or in multiple environments (Tables 3, 4). Based on the SoyBase database and the results of recent studies, 12 of the 22 common QTNs appeared to be near QTLs associated with PC in soybean that had been mapped in earlier studies (Lee et al., 1996; Brummer et al., 1997; Csanádi et al., 2001; Jun et al., 2008; Lu et al., 2013; Mao et al., 2013; Pathan et al., 2013; Hwang et al., 2014; Qi et al., 2014; Vaughn et al., 2014; Warrington et al., 2015): AX-157074676, AX-157594705, AX-157397239, AX-157489326, AX-157462104, AX-157506141, AX-157069070, AX-157357710, AX-157217990, AX-157570733, AX-157566978, and AX-157168337 (Table 4, bold text). This indirectly confirmed the accuracy of our QTN detection. The remaining ten QTNs in this study were new (Table 4, non-bold text). For this population, the detection of significant QTNs is not only a way to identify the genes related to PC, but can also identify good lines based on the superior allele information to support breeding high PC soybean.

Based on the 22 common QTNs detected here and their pathway annotation, we have identified 8 genes that may be related to protein anabolism (Table 7, bold text). *Glyma.03G100800* is intimately involved in the biosynthesis of amino acids, and the pentose phosphate pathway which it is involved in also indirectly affects the biosynthesis of proteins (Xu et al., 2018). *Glyma.10G207300*, *Glyma.14G081600*, and *Glyma.12G019300* are mainly involved in the proteasome pathway and work as protease to degrade proteins to small peptides and amino acids, so we think that these three genes are closely related to protein degradation. *Glyma.18G071100* may play an important role in protein anabolism; it participates in the process of glycosylation in endoplasmic reticulum, and the function of glycosylation is to enable proteins to resist the digestive enzymes, so as to protect protein from degradation (Jayaprakash and Surolia, 2017). *Glyma.12G112900* is a kind of riboflavin synthase and it participates in the biosynthesis of riboflavin, which plays an important role in energy metabolism including carbohydrate, protein and fat metabolism (Tuan et al., 2014; Zhao et al., 2014). *Glyma.18G071300* participates in RNA transport, and this process is essential in protein synthesis; as we know, it mainly carries amino acids into ribosomes and synthesizes proteins under the guidance of mRNA. *Glyma.18G028600* is a kind of translocation protein, and it mainly works in post-translational transport and post-translational modification in the process of protein synthesis, so it plays an important role in protein synthesis (Kwon et al., 1999). Based on these 8 genes, further work will be needed to determine which of these actually significantly affect PC in soybeans, and then to identify the target genes. This information can be used as the basis for further exploration of the gene network for the trait.

In recent years, GWAS has been widely applied to crop plants such as rice (Huang et al., 2010; Ma et al., 2016), maize (Tian et al., 2011), and soybean (Li et al., 2018), and the model mainly used for GWAS is mixed linear model (MLM). It belongs to single-locus GWAS, for which the screening criterion for significance is *P* = 0.05/*m* (where *m* is the number of markers) (Perneger, 1998). For a large number of SNPs, some important loci may be undetectable under this screening criterion.

In this study, we also used MLM to carry out the GWAS of soybean PC, with the calculation of population structure based on STRUCTURE 2.3.4 and the calculation of kinship and GWAS based on TASSEL 5.0. However, we did not detect any significant SNPs using the model. We believe that this was related to the screening criteria of single-locus GWAS and to the type of target trait as well as the population type. In light of this negative result, we tried changing the screening criteria, and replaced the strict Bonferroni correction with a less stringent false discovery rate (FDR) correction. The *q*-values were equal to the *P*-values adjusted with the Benjamini and Hochberg (2000) procedure; we used the cut-off of *q* < 0.05 and *P* < 0.0001as the threshold value to identify significant QTNs. Based on these screening criteria, we still did not find significantly associated SNP markers. To further reduce the stringency of the screening criteria, we next tried using a *P*-value of < 0.0001 directly as a cut-off without any corrections. With this criterion, a total of 15 SNP markers were identified with 1 cluster (bold text in Table 8). The range for the one cluster was 83.40 kb.

**Table 8:** Details of significant SNPs detected by MLM with screening critical *P* < 0.0001.

Environment	Chr	Physical position (bp)	Physical distance (kb)	*P*-value	No. of QTNs (MLM)^a^	No. of QTNs (multi-locis GWAS)^b^
E6	1	5,703,406		2.82E-05	1	1
E19	8	43,712,243		1.17E-04	1	1
E4	13	1,147,825		9.80E-05	1	0
E12	14	15,013,495		1.35E-04	1	0
E12	16	5,609,879		1.12E-04	1	0
E19	16	28,008,354		1.07E-04	1	2
E6	18	5,396,919		3.54E-05	1	0
E16	**18**	**6,590,065 - 6,673,462**	**83.40**	**6.01E-05 - 1.35E-04**	**8**	**11**
Total					**15**	**15**

*^a^Number of QTNs detected by MLM model. ^b^Number of QTNs detected by multi-locus GWAS methods.*

Because of the nature of single-locus methods, even if we identified these significant SNP markers under less stringent screening methods, we lack confidence about our ability to control for the false positive rate of results obtained without the correction for multiple tests. A previous study yielded a similar outcome to ours (Fang et al., 2017), so it seems evident that the single-locus method is not always suitable for detecting the genetic basis of complex traits.

To make up for the shortcomings of the above methods, multi-locus GWAS methods have recently been explored, including the five methods we used in this study: mrMLM (Wang et al., 2016), FASTmrMLM (Tamba et al., 2017), FASTmrEMMA (Wen et al., 2018), pLARmEB (Zhang J. et al., 2017), and ISIS EM-BLASSO (Tamba et al., 2017). Using the five methods, we detected a total of 19, 18, 12, 37, and 43 significant QTNs, respectively (Figure 3B and Supplementary Table S4). The differences in the numbers of QTNs detected by the five methods are presumed to be due to the different principles underlying the different methods: even though all five are two-stage combined approaches, they differ in the models and methods for screening and estimation. Because the main purpose of this study required the most accurate QTNs possible, we felt it desirable to take into consideration the results of all five methods, and took QTNs detected by multiple methods as the credible QTNs to use in further experiments. This practice adds an extra screening to the multi-locus GWAS approach and thus makes us more confident about the results.

Based on the data from the QTNs we detected, we found that the absolute values of QTN effects were all relatively low, in the range of 0.17–1.35, which indirectly explained why we could not detect significant QTNs by MLM with the standard Bonferroni correction. Thus, the greatest advantage of the multi-locus GWAS approach was its ability to find loci with relatively small effects. In addition, the multi-locus GWAS method was more suitable for the FW-RIL population used in this study than single-locus GWAS. This is because the single-locus GWAS method is generally based on SNPs for which there are only two alleles at one locus, meaning that the multi-allelic variation that exists at some genetic loci in the FW-RIL population cannot be detected. However, the multi-locus GWAS method overcomes this limitation: because it is based on a multi-locus and multi-allele model, it can identify genome-wide QTNs in a more comprehensive fashion along with the multiple alleles. This is the other reason that we were able to detect significant QTNs with the multi-locus GWAS method.

## Summary

Combining five multi-locus GWAS methods, we identified 22 common QTNs, including one stable expression QTN AX-157298785. Around these QTNs, 8 potential candidate genes were identified. Moreover, we selected elite lines for breeding higher seed protein content.

## Author Contributions

W-XL and HN conceived and designed the experiments. WL, ShiL, SX, JiZ, KZ, XL, YF, YW, JS, ZQ, and XT made major contributions to the field experiments and determination of quality traits. ShuL and ZT performed the genome sequencing. KZ, HN, and JuZ analyzed and interpreted the results. KZ and HN drafted the manuscript. All the authors contributed to the manuscript revision.

## Conflict of Interest Statement

The authors declare that the research was conducted in the absence of any commercial or financial relationships that could be construed as a potential conflict of interest.

## Footnotes

1 https://phytozome.jgi.doe.gov

2 http://www.bar.utoronto.ca

3 http://www.plexdb.org

4 http://www.kegg.jp

## Supplementary Material

FIGURE S1 The mean values of seed protein content for 144 lines and 4 parents across 20 environments.

FIGURE S2 Linkage disequilibrium (LD) decays in the four-way recombinant line (FW-RIL) population. **(A)** The LD decay rate was estimated as the squared correlation coefficient (*r^2^*) using all pairs of SNPs located within 10 Mb of physical distance. The dashed line in red indicates the position where *r^2^* dropped to half of its maximum value, and the dashed line in green indicates the position where *r^2^* dropped fast and then tended to flatten. **(B)** Enlarged display of the area in the purple frame in **(A)**.

TABLE S1 Summary of details of planting conditions in field experiments.

TABLE S2 Statistical characteristics of protein content in the parents and the FW-RIL populations grown in twenty environments.

TABLE S3 The correlation analysis between each pair of environment.

TABLE S4 Significant QTNs for seed protein content across 20 environments using the five multi-locus GWAS methods.

TABLE S5 The information of pathways of 17 genes annotated in the KEGG database.

TABLE S6 The information of orthologous protein families of 17 genes annotated in the KEGG database.
