# Supplementary figures and images for: Identification of QTNs Controlling Seed Protein Content in Soybean Using Multi-Locus Genome-Wide Association Studies
Source: Front Plant Sci. 2018 Nov 21;9:1690. doi: 10.3389/fpls.2018.01690 (PMC6258895; doi:10.3389/fpls.2018.01690)

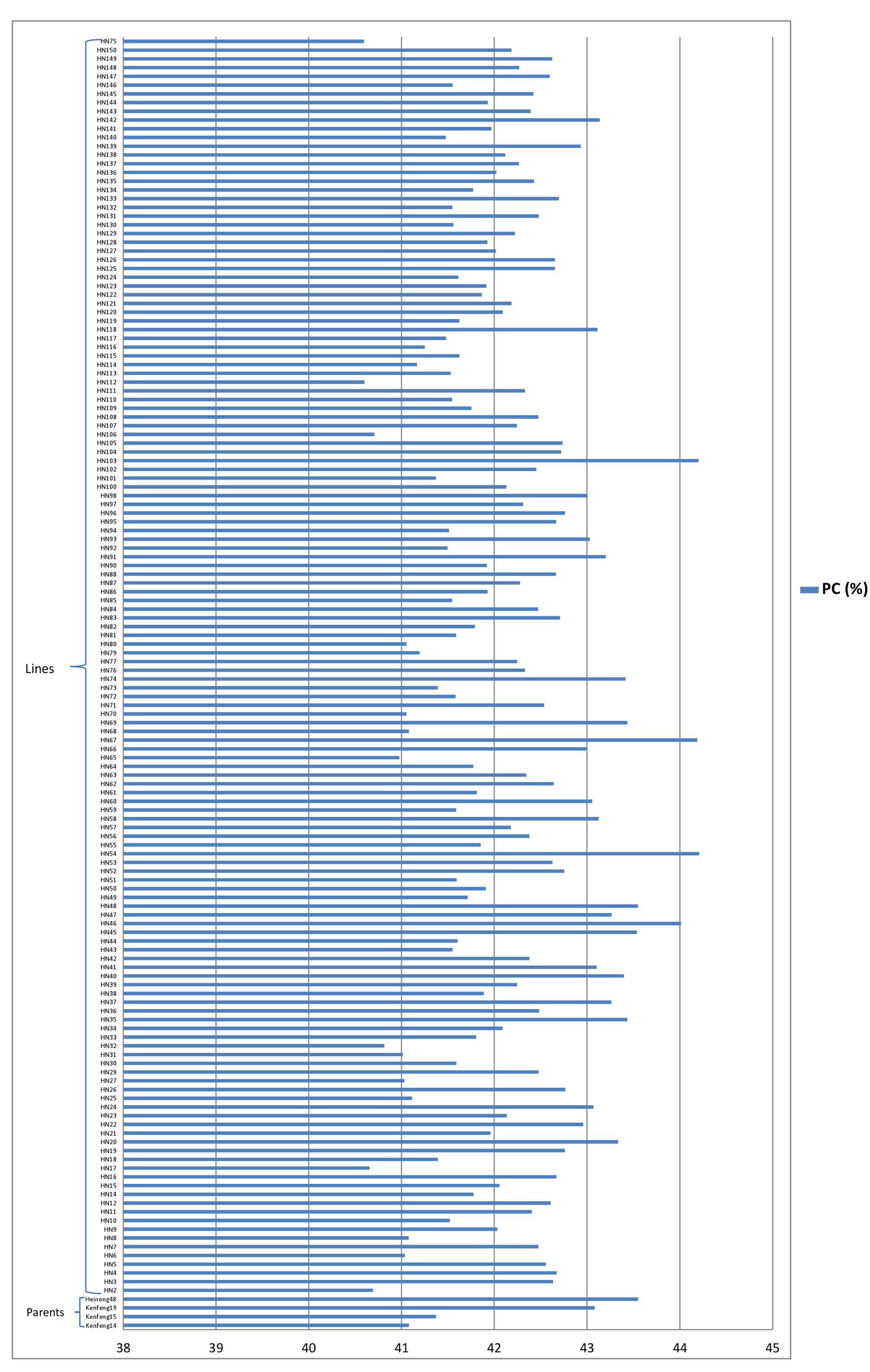

Supplement: FIGURE S1 — The mean values of seed protein content for 144 lines and 4 parents across 20 environments. [file Image_1.JPEG]

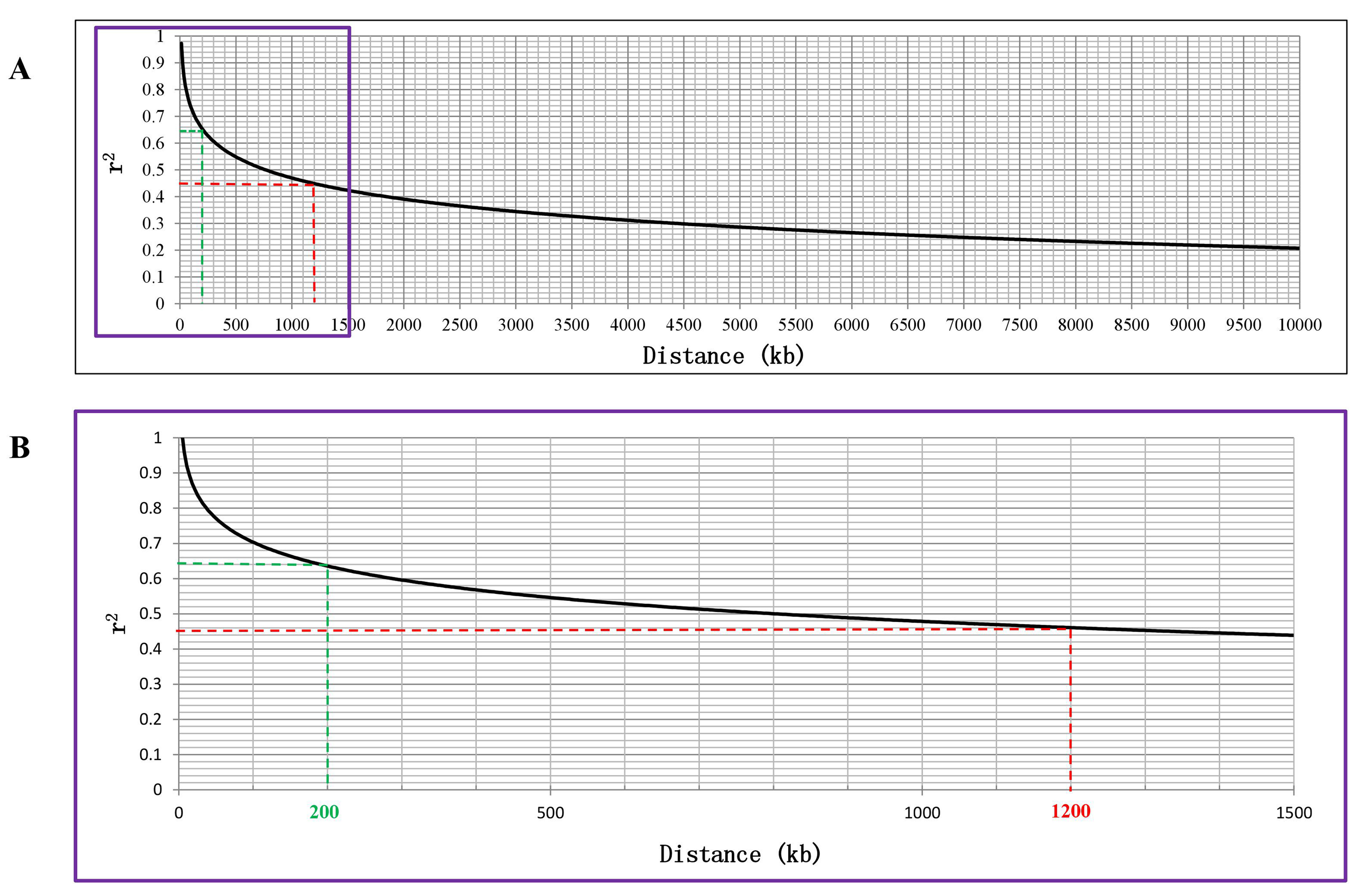

Supplement: FIGURE S2 — Linkage disequilibrium (LD) decays in the four-way recombinant line (FW-RIL) population. (A) The LD decay rate was estimated as the squared correlation coefficient (r2) using all pairs of SNPs located within 10 Mb of physical distance. The dashed line in red indicates the position where r2 dropped to half of its maximum value, and the dashed line in green indicates the position where r2 dropped fast and then tended to flatten. (B) Enlarged display of the area in the purple frame in (A). [file Image_2.JPEG]
